# Non-Motor Disorders in Parkinson Disease and Other Parkinsonian Syndromes

**DOI:** 10.3390/medicina60020309

**Published:** 2024-02-11

**Authors:** Anastasia Bougea, Efthalia Angelopoulou

**Affiliations:** Department of Neurolgy, Eginition Hospital, School of Medicine, National and Kapodistrian University of Athens, 11528 Athens, Greece; angelthal@med.uoa.gr

## 1. Introduction

Parkinsonism is an umbrella term that refers to multisystemic neurodegenerative disorders characterized by a broad spectrum of motor and non-motor symptoms (NMSs) [1]. The parkinsonian syndromes include idiopathic Parkinson disease (PD), progressive supranuclear palsy (PSP), multiple system atrophy(MSA), corticobasal degeneration (CBD), and vascular Parkinsonism (VaP), among other rare presentations of parkinsonism [2]. Neuroanatomically, NMS may be subdivided into cortical manifestations (psychosis and cognitive impairment), basal ganglia symptoms (impulse control disorders, apathy, and restlessness or akathisia), brainstem symptoms (depression, anxiety, and sleep disorders), peripheral nervous system disturbances (orthostatic hypotension (OH), constipation, and pain), and sensory disturbances [3]. NMSs are often overlooked by neurologists and dismissed by patients, making their management difficult, with a major burden for patients and caregivers [4]. Unfortunately, there are limited data about the prevalence of NMSs, their neurobiology, their potential biomarkers, their monitoring, and their treatment. It is, therefore, essential that researchers and clinical neurologists comprehensively address the factors related to NMS in order to improve quality of life for PD patients.

This Special Issue of *Medicina* entitled “Non-motor Disorders in Parkinson Disease and Other Parkinsonian Syndromes” includes articles investigating the role of NMS in disease pathogenesis in addition to their potential for monitoring and designing mechanism-based therapies.

## 2. An Overview of Published Articles

A single-center study from Latvia [5] explores the impact of NMSs on the quality of life of 43 PD patients with different motor phenotypes (TD: tremor dominant; and PIGD: postural instability/gait difficulty). The most common NMS of PD patients were fatigue (95.3%), sleep disturbance (83.7%), daytime sleepiness (83.7%), pain and other sensations (81.4%), urinary problems (69.8%), constipation (65.1%), depressed mood (64.3%), lightheadedness upon standing (58.1%), apathy (57.1%), anxiety (42.9%), dopamine dysregulation syndrome (14.3%), and hallucinations and psychosis (7.1%). NMSs such as fatigue, apathy, sleep problems and daytime sleepiness, pain, and disturbances in gastrointestinal and cardiovascular function, were associated with a poorer health-related quality of life in PIGD compared with TD patients. Thermoregulatory and pupillomotor symptoms also significantly affect PD patients’ well-being.

Gulluoglu et al. [6] found visuospatial deficits in patients with PD and mild cognitive impairment (MCI) due to the decrease in brain volumes. Specifically, among PD-MCI patients, there was a more noticeable decline in white matter volume based on volumetric Magnetic Resonance Imaging compared to the localized loss of gray matter volume.

Impulse Control Disorders (ICDs) including pathological gambling, hypersexuality, compulsive eating, compulsive buying, and other related behaviors are NMSs of PD. A prospective study by Toś et al. [7] found a prevalence of 27.41% in Polish PD patients, which is comparable to other European populations. The risk factors for developing ICDs include a longer duration of the disease, motor complications, sleep disorders, and the use of DA and L-dopa. 

For the first time, Melnikova et al. [8] showed impaired abstraction tasks in 52 patients with Idiopathic Cervical Dystonia with severe depression as a major neuropsychiatric co-factor. Although not statistically significant, a slightly higher MoCA scale score was reported in cervical dystonia patients with clinically insignificant depressive signs.

The retrospective study of Lee et al. [9] measured the peak cough flow (PCF) of 219 patients with PD who underwent a videofluoroscopic swallowing testing. A novel finding was that a PCF value ≤153 L/min was associated with an increased risk of aspiration. These results underlined the low PCF as a risk factor for aspiration in patients with PD.

The meta-analysis conducted by Angelopoulou et al. [10] demonstrated a positive association of PD among lifetime migraineurs. PD patients suffering from any headache had a lower motor unified Parkinson’s disease rating scale (UPDRS) score (503 PD patients; SMD −0.39; 95% CI −0.57 to −0.21) compared to PD patients not reporting headache. Similar findings were reported by three studies with data on the TTH-PD relationship (high prevalence, positive association when examined prospectively, and an inverse relationship 12-monthprevalence).

## 3. Conclusions

This collection of the papers included in this Special Issue accumulates diverse aspects of NMSs in PD and related disorders, highlighting their importance in the field of movement disorder research. The diversity of results arising from the research presents a major methodological challenge for evaluating and evidencing the impact of NMSs in the quality of life of these patients.

The prevalence of NMS in PD patients from Latvia is in line with previous studies investigating diverse nationalities [5]. Moreover, by evaluating the impact of NMSs on distinct motor phenotypes, this study may trigger more research for the future personalized therapy of NMSs in PD patients. MCI is an underestimated NMS that frequently progresses to dementia in PD patients. To address this issue, Güllüoğlu et al. [6] suggested abnormal neuropsychological tests and brain volumetrics in PD-MCI patients as pretests of transition to dementia. Given the relatively limited sample size, larger-scale prospective studies are needed to confirm these findings. Apart from PD, depression may not only be a disabling NMS in other hyperkinetic movement disorder such as cervical dystonia but may also be associated with specific cognitive tasks, tested by validated neuropsychological scales [8]. Aspiration may also be a life-threatening NMS for patients with PD. Despite the moderate sensitivity (73.06%) and the specificity (51.06%), the low PCF could be proposed as a potential biomarker for screening for aspiration in patients with PD [9]. Despite the high prevalence of migraine across various PD cohorts, the potentially higher risk of incident PD among migraineurs in cohort studies was not verified (and even reversed) in sensitivity analyses [10]. This could be partially attributed to the potential benefit of PD on the migraine headache process and answers whether the analysis pooling all studies yields an overall non-significant result; however, this meta-analysis provides valuable knowledge to neurologists in determining the headache profiles of PD patients and treatment options.

Future studies should include longitudinal design, larger homogeneous samples (genetic types and nationality), and a longer follow-up. There is an undeniable need to apply validated diagnostic criteria, validated biomarkers, and standardized tests to evaluate the NMSs of PD. Thus, the current studies presented in this Special Issue should be seen not only as the novel aspects of NMS carried out by different methodologies, but also as a stimulant for new investigations.
